# Investigating and comparing the dimensions of worry of Iranian primiparous women in each trimester of pregnancy

**DOI:** 10.1186/s40001-023-01258-5

**Published:** 2023-08-16

**Authors:** Foruzan Mirzaee, Seyedeh Batool Hasanpoor-Azghady, Leila Amiri-Farahani

**Affiliations:** Department of Midwifery and Reproductive, Nursing and Midwifery Care Research Center, School of Nursing and Midwifery, Iran University of Medical Sciences, Rashid Yasemi St., Valiasr St., Tehran, 1996713883 Iran

**Keywords:** Worry, Pregnancy, Primiparous, Iranian women

## Abstract

**Background:**

Pregnancy and childbirth are considered natural events in the life cycle of women. However, it is also a stressful experience along with physiological and psychological changes. Therefore, it is important to study the dimensions that cause more worry in each of the pregnant trimesters. This study aimed to determine and compare the dimensions of worry of Iranian primiparous women in each trimester of pregnancy.

**Methods:**

This cross-sectional study was conducted on 300 primiparous women (*n* = 100 in each trimester) referred to seven health centers affiliated with the Iran University of Medical Sciences, Tehran, Iran. The sampling was multistage. We collected data from a demographic and fertility questionnaire and the Cambridge Worry Scale (CWS).

**Results:**

The mean score of worry during the entire pregnancy was 28.16. The mean and standard deviation of the worry score in the first trimester was (27.35 ± 12.22). The second trimester was (27.80 ± 12.53) and the third trimester was (29.34 ± 11.11). The highest mean score of worry in the first and third trimmers was the dimension of own health. The second trimester was the dimension of socio-medical. The lowest mean score of worry in all trimmers was the dimension of relationships. Among CWS-related items, the highest mean score of worry in the first trimester was giving birth (3.34) and the possibility of miscarriage (3.22). In the second trimester was the possibility of going into labour too early (3.3) and the possibility of miscarriage (3.12), and in the third trimester was the possibility of going into labour too early (3.33) and giving birth (3.27). The lowest mean score of worry in all three trimesters was related to problems with the law.

**Conclusion:**

pregnancy worry in the third trimester was more than the other two trimesters, and worrying about own health was the most important dimension of worry for pregnant women. Paying attention to the dimensions of worry of pregnant women helps design appropriate interventions to increase the mental and physical health of pregnant women.

## Background

Pregnancy is one of the most important stages of a woman’s life. Although it may be a positive experience for most pregnant women, it is often a stressful experience along with physiological and psychological changes [1]. Tensions and worries in people can lead to anxiety if left untreated [2]. Anxiety and concern can take the form of illness and affect the mental health of the mother and child [3]. The worry is also associated with an increased risk of social and occupational disorders, increased use of health services and physical problems in society [4, 5]. During pregnancy, depression, anxiety, stress, and severe concern have adverse consequences for the mother, family and child’s health and well-being in the short and long term [6]. A study in Ireland showed that 43% of women had concern and fear for their childbirth [7]. Osborne et al. reported high antenatal worry during pregnancy is one of the important predictors of postpartum depression [8]. The results of a study in Iran revealed that the level of concern during pregnancy was moderate for 62.4% of the subjects and severe for 18.4 of them [9]. Pregnancy concerns are more in developing countries or countries that are in a state of economic turmoil [5].

Fenwick et al. reported that contributing factors of concern and fear of pregnancy in primiparous women include; thoughts of having a disabled or abnormal child, being alone during pregnancy, doing something wrong during pregnancy that may adversely affect the fetus, and having labour pains. Other factors that can increase the level of concern include misperception of social support by mothers, and physical and mental unreadiness for childbirth [10]. In a qualitative study on 20 pregnant women, three fathers and three experienced midwives, the main concerns of pregnant women were usually related to being a good mother, having a healthy delivery, life changes after childbirth, infant’s health threatened marital life, and inability to raise and care for the child [11]. High levels of concern in the late pregnancy period may adversely affect women’s ability to give birth [12]. Negahban and Ansari referred to the fear of pain (68%) and fetal injury (30%) as the most common concerns of women during pregnancy [13]. While Borghei et al. found that fetal health was the most important concern of participants during their pregnancy [11]. Although it is normal to have a low level of worry during pregnancy, a high level of concern can increase stress and be considered a pathological factor. Studies have shown that pregnancy concerns not only affect the mental health of pregnant women but can also affect the outcome of labour and cause complications such as preterm labour, prolonged labour, cesarean delivery, low birth weight, postpartum depression, and later development of the newborn [14–18].

Green et al. classified into worries of pregnancy four dimensions: socio-medical, socio-economic, own health and relationships [19]. The results of some studies that examined Iranian pregnant women's satisfaction with prenatal care, especially the childbirth experience and how to care and support women during the labor process, showed that the level of satisfaction was moderate [20, 21]. The unpleasant experience of childbirth, such as frequent internal examinations and the lack of emotional support for women [22, 23] when it is available to pregnant women in different ways, increases their worries to the extent that they tend to have a cesarean section. These results indicate the need to investigate the dimensions of pregnant women's concerns. On the other hand, the economic challenges and problems that Iran has found in the last five or six years [24] have made the socio-economic dimension (one of the dimensions of concern during pregnancy) more prominent. Also, perinatal mental health research has focused more on the postpartum period [25]. Some of the studies that examined pregnancy concerns were qualitative [11] that were not generalizable. Quantitative studies either selected a gestational age range [25–27] or were performed in one or two trimesters of pregnancy [17, 28]. While the present study investigated the dimensions of the worry of primiparous women in each trimester of pregnancy.

Another noteworthy point is that the causes of pregnancy worry may vary in different cultures. For example, the concern about relationships, which is important in developed countries [17], has little importance in developing countries like Iran [28]. For this reason, understanding the dimensions of concern in each trimester of pregnancy and comparing them in different trimesters can provide a more comprehensive view to experts who work on aspects of women's health. Based on this, appropriate interventions can be used to improve and develop mental health services in each trimester of pregnancy. For this purpose, this study aimed to determine and compare the dimensions of worry of Iranian primiparous women in each trimester of pregnancy.

## Methods

This cross-sectional study was conducted on 300 primiparous women (*n* = 100 in each trimester) referred to seven health centers affiliated with the Iran University of Medical Sciences. This university covers seven districts in Tehran (the capital of Iran). In the Iranian health care system, a midwife visits a pregnant woman eight times in health centers. If necessary, she is referred to a general practitioner or obstetrician. Each woman can also decide individually to go to a private medical center. The sample size was estimated at 289 subjects with a 95% confidence interval, 80% test power, a standard deviation of *σ* = 8.5, and accuracy of *d* = 1 through the following formula.

$$n\, = \,\frac{{z_{1 - {\raise0.7ex\hbox{$\alpha $} \!\mathord{\left/ {\vphantom {\alpha 2}}\right.\kern-0pt}\!\lower0.7ex\hbox{$2$}}}^2 \, \times \,\sigma^2 }}{d^2 }\,;\,n = \frac{1.96^2 \, \times \,72.25}{{1^2 }}\, = \,289$$

We used the study of kordi et al. for the standard deviation of worry. They collected data with CWS [29]. Because in society, the ratio of primiparous women in each trimester was almost equal; with equal allocation, we selected 100 primiparous women for each trimester. The sampling was done by the multi-stage method. First, a health center was randomly chosen from each district then the eligible individuals were determined as a quota in each center. Next, continuous sampling was conducted at each center between November 2018 and August 2019.

We asked pregnant women who were  ≥ 18 years old to participate in the study. Their other inclusion criteria were; having the ability to read and write, lack of stressful events in the last 6 months, not using drugs or any medications related to mental disorders, having no chronic systemic disease, having no high-risk pregnancy, having a single pregnancy, and the lack of abnormality and pathological problems in the fetus based on ultrasonography. During the sampling period, we examined 327 participants, of which 24 did not have the study inclusion criteria. Refusal to participate was very low (about 1%).

Data collection tools included the demographic and fertility questionnaire and the Cambridge Worry Scale (CWS) developed by Green et al. The CWS is a self-administered tool for assessing the content and extent of worries in pregnancy [19]. It includes four dimensions of socio-medical (Items 10, 11, 12, 13, 15), socio-economic (Items 1, 2, 3, 8, 14), own health (Items 6, 7, 9, 16, 17), and relationships (Items 4 and 5). It contains items concerning such issues as the baby's health and giving birth. Depending on the pregnancy week, additional context-specific items can be added or removed from the tool as needed. This scale consists of 17 items. Each item is scored on a six-point Likert-type scale ranging from not a worry (0) to major worry (5). The CWS scale can be used throughout pregnancy. In the first trimester, the item “the possibility of going into labour too early”, and in the third trimester, the item “the possibility of miscarriage” were removed. Therefore, the scale of the first and third trimester have 16 items. The scores ranged from 0 to 80 in the first and third trimesters and 0–85 in the second trimester. On this scale, a higher score reflects higher worries. At the end of the scale is an open-ended question. The question enquires about worries not listed on the scale [19]. In Iran, kordi et al. reported internal consistency of 0.9 for this tool using Cronbach’s alpha coefficient [29].

The Ethics Committee of Iran University of Medical Sciences (Tehran, Iran) confirmed the current research project with the ethics code IR.IUMS.REC.1397.545. After obtaining sampling permission from the Iran University of Medical Sciences, we started the sampling at the health centers. When pregnant women went to health centers for prenatal care, we first identified the eligible participants among them. Then we explained the purpose of the research and the principle of confidentiality to them and obtained their written consent.

We used descriptive statistics for the demographic, fertility characteristics, and the dimensions and items of the CWS. To investigate the relationship between demographic variables and fertility with worry, independent t-test and one-way ANOVA were used in SPSS software version 22. The significance level for all tests was *P* < 0.05.

## Results

The age range of women was between 18 and 42 years with a mean and standard deviation of 26.39 ± 5.32. The results showed that the mean and standard deviation of worry in the entire pregnancy was 28.16 ± 11.39. More information about the demographic and fertility characteristics of the subjects is presented in Table 1.

**Table 1:** Demographic and fertility characteristics and their relationship with worry in primiparous women (*n* = 300)

Characteristics	*N* (%)	Mean (SD)	*P*-value
Woman’s age			0.95*
< 21	64 (21.3)	27.59 (12.42)	
21–25	67 (22.7)	28.71 (13.27)	
26–30	105 (35)	28.29 (10.8)	
> 30	63 (21)	27.95 (12.11)	
Woman’s education			0.08*
< High school	59 (19.7)	29.85 (12.8)	
High school	100 (33.3)	29.47 (11.99)	
Academic	141 (47)	26.53 (11.44)	
Woman’s occupation			0.32**
Housewife	233 (77.3)	28.53 (12.1)	
Employed	67 (22.3)	26.9 (11.78)	
Abortion history			0.48**
Yes	52 (17.3)	29.23 (12.48)	
No	248 (82.7)	27.94 (11.86)	
Pregnancy			0.25**
Wanted	269 (89.7)	27.9 (11.87)	
Unwanted	31 (10.3)	30.48 (12.72)	
Wanted and unwanted baby’s gender			0.35*
Wanted	161 (53.7)	28.22 (11.35)	
Unwanted	42 (14)	30.33 (12.93)	
Uncertain	97 (32.3)	27.13 (12.51)	

^*^One-way ANOVA

^**^Independent t-test

Table 1 shows that there was no association between demographic and fertility variables with worry in primiparous women. The mean and standard deviation of worry and its dimensions in each pregnancy trimmer is also presented in Table 2.

**Table 2:** Means and standard deviations of subscales of CWS in in each trimester of pregnancy (n = 300)

Dimensions of CWS	Pregnancy trimester								
	First trimester (*n* = 100)	Achievable score	Obtained score	Second trimester (*n* = 100)	Achievable score	Obtained score	Third trimester (*n* = 100)	Achievable score	Obtained score
	Mean (SD)			Mean (SD)			Mean (SD)		
Socio-medical	9.95 (4.75)	0–25	1–19	9.74 (5.19)	0–25	3–22	10.04 (4.45)	0–25	3–24
Socio-economic	7.06 (5.24)	0–25	1–20	8.10 (4.88)	0–25	3–24	7.83 (4.93)	0–25	3–24
Own health	8.76 (4.73)	0–20	2–20	8.12 (4.21)	0–25	3–23	9.43 (4.03)	0–20	2–20
Relationships	1.58 (2.37)	0–10	0–5	1.81 (2.53)	0–10	0–5	2.04 (2.60)	0–10	0–7
Total score CWS	27.35 (12.22)	0–80	7–63	27.80 (12.53)	0–85	8–64	29.34 (11.11)	0–80	8–64
Mean based on one hundred of total score CWS	34.18			34.75			36.67		

Table 2 shows that the highest mean score of worry (from 100) was related to the third trimester (due to the difference in the number of items in the first and third trimesters, and in the second trimester, the mean score was calculated based on 100).

**Fig. 1:**
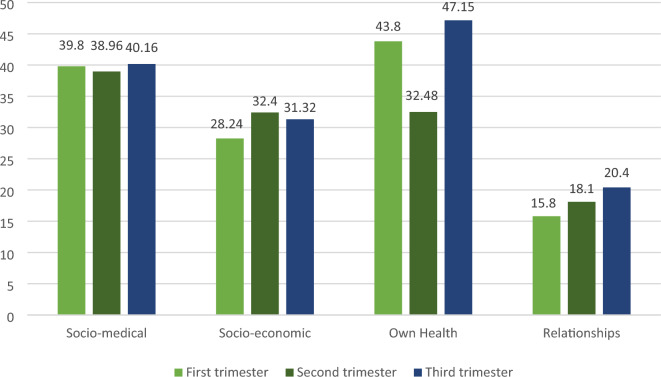
Chart of dimensions of CWS in all trimesters of pregnancy (*n* = 300)

Figure 1 shows the highest mean score of worry in the first and third trimesters was the dimension of own health and the second trimester was the socio-medical dimension. The lowest mean score of worry in all trimmers was the dimension of relationships (due to the difference in the number of items in the CWS dimensions, the mean scores in Fig. 1 presented based on 100).

The mean and standard deviation of GWS items in each of the pregnancy trimmers are presented in Table 3.

**Table 3:** Means and standard deviations of items of CWS in all trimesters of pregnancy (n = 300)

	Items of CWS	First trimester	Second trimester	Third trimester
		Mean (SD)	Mean (SD)	Mean (SD)
1	Your housing	2.18 (2.04)	2.44 (1.95)	2.24 (1.94)
2	Money problems	2.17 (1.69)	2.83 (1.63)	2.74 (1.59)
3	Problems with the law	0.43 (0.86)	0.55 (1.14)	0.50 (1.04)
4	Your relationship with your husband/partner	0.77 (1.32)	0.80 (1.33)	0.90 (1.34)
5	Your relationship with your family and friends	0.81 (1.18)	1.01 (1.43)	1.14 (1.42)
6	Your own health	1.43 (1.44)	1.29 (1.58)	1.55 (1.52)
7	The health of someone close to you	2.04 (1.70)	1.53 (1.44)	2.23 (1.54)
8	Employment problems	1.54 (1.86)	1.43 (1.72)	1.53 (1.79)
9	The possibility of something being wrong with the baby	2.07 (1.61)	2.18 (1.46)	2.23 (1.71)
10	Going to hospital	2.24 (1.60)	2.17 (1.60)	2.16 (1.54)
11	Internal examinations	1.60 (2.20)	2.33 (1.57)	2.22 (1.49)
12	Giving birth	3.34 (1.51)	2.88 (1.61)	3.27 (1.53)
13	Coping with the new	1.23 (1.32)	1.33 (1.41)	1.33 (1.58)
14	Giving up work (if applicable)	0.74 (1.39)	0.88 (1.37)	0.82 (1.47)
15	Whether your partner will be with you for the birth	0.94 (1.29)	1.03 (1.44)	1.06 (1.47)
16	The possibility of miscarriage	3.22 (1.67)	3.12 (1.68)	–
17	The possibility of going into labour too early	–	3.30 (1.70)	3.33 (1.69)

## Discussion

The results of this study aimed to determine and compare the dimensions of worry of Iranian primiparous women in each trimester of pregnancy, showed that the mean score of worry in the entire pregnancy was 28.16. Comparing the mean score of worry in each trimester, the mean score in the third trimester was higher than the other two trimesters of pregnancy. Regarding the dimensions of worry in pregnancy, the results showed that the highest mean score of worry in the first and third trimesters was the dimension of own health. The second trimester was the socio-medical dimension. The lowest mean score in all trimmers was the dimension of the relationships. Regarding CWS items, the highest mean score in the first trimester was the items of "giving birth" and "the possibility of miscarriage", in the second trimester, the items of "the possibility of going into labour too early" and "the possibility of miscarriage", and in the third trimester was the item of "the possibility of going into labour too early". The lowest mean score of worry in all three trimesters was the item of "problems with the law".

In a study conducted by Malakouti et al. on 465 pregnant women in the second and third trimesters, the mean and standard deviation of concern score was 11.10 ± 6.4 in the dimension of socio-medical, 5.40 ± 4.6 in the dimension of socio-economic, 7.20 ± 4.9 in the dimension of own health, and 2.00 ± 2.40 in the dimension of relationships [28]. The mean score of worry in each trimester has not been mentioned separately in this study. The results of this study are in line with the findings of the present study regarding the second trimester that the highest mean score of worry in pregnancy was the socio-medical dimension, and the lowest mean score was the dimension of relationships. Another study in Spain on 285 pregnant women in the first and third trimesters showed the mean score of worry was 2.34 in the dimension of socio-medical, 4.37 in the dimension of socio-economic, 12.58 in the dimension of own health and 3.99 in the dimension of relationships [17]. The results of this study are consistent with the finding of the present study regarding the highest mean score of worry in the dimension of own health, but the mean scores of two socio-medical and socio-economic dimensions were lower in the above study than the mean scores of similar dimensions in the present study. Also, the mean score of the relationships dimension in the above study was higher than the mean score of the relationships dimension in the present study. The difference between the two studies could be due to the differences in culture of the two study communities and the parity of pregnant women because our study was conducted only on primiparous women, but the above study was on both primiparous and multiparous women.

In a study, Gourinti et al. examined the worry of 163 Greek pregnant women with the gestational age of 11–26 weeks, of whom 46% were primiparous. The tool used in this study CWS was similar to the one used in the present study. The results of this study showed the mean score of worry was 26 and the highest mean scores of worries were items 2 (money problem), 9 (the possibility of something wrong with the baby) and 12 (giving birth), respectively [26]. The mean score of concern in this study was slightly lower than the mean total score of worry in our study. Also, in the present study, the highest mean score of concern was related to items 2, 12, 16 (the possibility of miscarriage) and 17 (the possibility of going into labour too early). This difference may be because the subjects in our study were only primiparous women, while in the mentioned study, the subjects were both primiparous and multiparous women. On the other hand, the present study was performed in all pregnancy trimmers, while the above study did not include the third trimester.

The results of the present study are consistent with the findings of the study by O’Connell et al. as both studies show the increasing worry of pregnant women as the time of delivery approaches [30]. In our study, the highest mean score of worry was related to the third trimester.

The results of a study conducted on 344 German pregnant women (200 of whom were primiparous) showed that the highest mean score of worry was related to the items of giving birth (2.26), the possibility of something wrong with baby (1.99), coping with the new baby (1.57), going to the hospital (1.29) and going to labour too early (1.28). The results of this study were consistent with our study, except that the mean score of worry in each of these items in the present study was slightly higher than in mentioned study. This difference may be because our study subjects are all primiparous women, but the German pregnant women are both primiparous and multiparous. On the other hand, Iranian pregnant women often do not attend training classes on pregnancy and childbirth, but German pregnant women participating in this study were all selected from antenatal training classes, which causes them to be less worried compared to Iranian women. The lowest level of worry in German pregnant women was related to the items of Your relationship with your family and friends (0.45), Your relationship with your family and friends (0.42), problems with the law (0.15) and internal examinations (0.45), [31]. The same items except “internal examinations" had the lowest mean score in our study. Another study conducted on 132 pregnant women with a gestational age of 11–14 weeks at a public hospital in Greece showed that the highest mean score of worry was related to the items of the possibility of something wrong with the baby (2.9), giving birth (2.8), money problems (2.6) and the possibility of miscarriage (2.5) [27]. Also, the lowest mean score of worry was related to the items of problems with the law (0.3), your relationship with your family and friends (0.5), your relationship with your husband/partner (0.8) and whether your partner will be with you for the birth (0.8). The findings of this study are consistent with our study in the first trimester [27].

Singnal et al. examined the worry, anxiety and depression of 1,144 women with a gestational age of 35–37 weeks in New Zealand. Pregnant women were both primiparous and multiparous. The tool used for measuring worry in this study was the Brief Measure of Worry Scale (BMWS) with eight items. A worry of higher than 12 is considered a disturbing worry in this tool. The results of this study showed that the mean score of worry of the participants was higher than 12, which was considered a disturbing concern [25]. The mean score of worry in this study was higher than the median of the study tool, but in our study, the mean score of worry was lower than the median of the tool used in our study. This difference in the mean score of worry could be due to the difference in sampling time as in the above study worry has been measured in the last 2 weeks of pregnancy, while in our study, it was measured during the entire third trimester, which is 14 weeks.

Our study indicated that the mean score of worry was not statistically significant in different age groups of subjects. Contrary to our findings, other studies have reported that worry during pregnancy is higher in younger women [17, 32, 33]. Perhaps this difference is related to our subjects that all were primiparous and 80% of them were under 30 years old. In our study, although the mean worry score was lower in women with academically educated and employed women and higher in women with a history of abortion, unwanted pregnancy, and unwanted fetal gender, these differences were not statistically significant. Barjasteh and Moghaddam did not find a significant association between a history of abortion and pregnancy concerns [34] which was consistent with our result. Contrary to our findings, some studies have reported that pregnant women who had a previous abortion showed more concerns, especially for fetal health [17, 26]. Zhou et al. Also stated that worry and anxiety are more in unplanned pregnancies [35].

## Strengths and limitations of the study

### Strengths

We randomly selected one health center from each of the seven regions covered by the Iran University of Medical Sciences, which were in different parts of Tehran (Iran's capital), to make the study results more generalizable. Also, we checked pregnancy worries in each trimester so that it is possible to compare them for the health team. In Iran's health centers, the health team that provides services to pregnant women usually do not investigate their worries, therefore, pregnant women do not know that they can get help from their health team regarding this item. The results of the present study showed that the highest mean score of worry in the first and third trimesters was the dimension of own health; It is recommended that the examination of women's pregnancy worries in each trimester is one of the main items of their pregnancy file so that the necessary training can be given to have a healthier and safer pregnancy.

### Limitations

Perhaps one of the reasons for the differences in the results of the studies is related to variables such as the difference in the number of visits that a woman receives during pregnancy, differences in the quality of care and training, differences in people providing health care services (such as midwives, general practitioners, gynecologists or a group of these people), and hearings of pregnant women from those around and multiparous women about pregnancy and childbirth. Each of the listed variables can affect the mean worry of some CWS items. Conducting an extensive study that could cover all of the variables listed was not in the form of a master's thesis. For this reason, in our study, these variables were not examined. Therefore, researchers suggest that future studies pay attention to these variables.

In our study, the subjects were all selected from health centers, which may limit the generalizability of the results. However, health centers provide the main health services for the Iranian population.

## Conclusion

The results of the present study showed the worry of pregnant women was at the highest in the third trimester of pregnancy and also, the highest mean score of worry in the first and third trimesters was the dimension of own health and in the second trimester was the socio-medical dimension. Also, the lowest mean score in each trimester was the dimension of relationships. The highest mean score of worry in the first trimester was the items of "giving birth" and "the possibility of miscarriage", in the second trimester the items of "The possibility of going into labour too early" and "the possibility of miscarriage" and in the third trimester was related to the item of "the possibility of going into labour too early". It is important to be aware of the causes of pregnancy worries because worry may cause distress and complications in pregnant women and their newborns. Paying attention to causes of pregnancy worries provides a more comprehensive view for planners and providers of health and psychological services so that they could design intervention programs to reduce worry during pregnancy and increase the mental health of pregnant women.

## Data Availability

The datasets generated and/or analyzed during the current study are not publicly available due [REASON WHY DATA ARE NOT PUBLIC] but are available from the corresponding author on reasonable request.

## Abbreviation

CWS: Cambridge Worry Scale
